# Supplementary material for: Morphospace saturation in the stem-gnathostomes pteraspidiformes heterostracans: an early radiation of a ‘bottom’ heavy clade
Source: PeerJ. 2018 Jul 20;6:e5249. doi: 10.7717/peerj.5249 (PMC6055588; doi:10.7717/peerj.5249)
Supplement: Appendix S2 [file peerj-06-5249-s002.docx]

**Appendix 2. Character list**

**Discrete Characters**

Character list as in Randle & Sansom (2017). See the original for character source and argumentation.

**Character 1**. Pineal plate (separate plate): **(0)** absent, **(1)** present

**Character 2**. Pineal opening (macula): **(0)** uncovered, **(1)** covered.

**Character 3**. Pineal plate enclosed by dorsal plate: **(0)** absent, **(1)** present. Pineal plate enclosed by dorsal plate is condition seen in Anchipteraspididae.

**Character 4**. Pineal plate morphology: **(0)** triangular, **(1)** quadrangular, **(2)** circular/ovate, **(3)** flat topped ovate.

**Character 5**. Quadrangular pineal plate morphology, **(0)** rectangular, **(1)** anterior and posterior edge convex, **(2)** anterior edge concave, posterior edge convex, **(3)** posterior edge convex.

**Character 6**. Pineal-orbital belt: **(0)** absent, **(1)** present. Present when the orbital and pineal plates are in contact with each other separating the dorsal and rostral plates.

**Character 7**. Pineal-orbital plate contact: **(0)** point contact (very small contact, just point to point), **(1)** side contact (contact majority of pineal width).

**Character 8**. Rostral plate (separate plate): **(0)** absent, **(1)** present.

**Character 9**. Pre-oral surface (ornamented medial area on the ventral side of rostral plate): **(0)** absent, **(1)** present.

**Character 10**. Pre-oral field (unornamented area on the ventral side of rostral plate prior to the ascending lamella): **(0)** absent, **(1)** present.

**Character 11**. Ornamentation of pre-oral surface: **(0)** transverse **(1)** variable orientation of ridges.

**Character 12**. Anterior shape of rostral plate: **(0)** rounded, **(1)** rounded to a point, **(2)** truncated/ concave, **(3)** triangular.

**Character 13**. Orbital notch in rostral plate: **(0)** absent, **(1)** present.

**Character 14**. Orbital notch in the rostral plate: **(0)** rounded, **(1)** angular.

**Character 15**. Rostral plate pineal plate contact: **(0)** concave contact, **(1)** no notch, **(2)** convex notch.

**Character 16**. Pair of orbital plates (separate plates): **(0)** absent, **(1)** present.

**Character 17**. Possession of fused orbito-cornual plate: **(0)** absent, **(1)** present. Fused orbit-cornual condition seen in the Anchipteraspididae.

**Character 18**. Dorsal position of the orbits: **(0)** absent, **(1)** present. Coded as present if the entire orbital opening is seen in dorsal view.

**Character 19**. Delimitation of the branchial opening: **(0)** branchial plate & dorsal plate, **(1)** branchial plate & cornual plate, **(2)** branchial plate, dorsal plate & cornual plate, **(3)** branchial plate & fused orbito-cornual plate.

**Character 20**. Branchial opening position on the dorsal plate: **(0)** posterior end of dorsal plate, **(1)** lateral side of dorsal plate.

**Character 21**. Pair of cornual plates (separate plates): **(0)** absent, **(1)** present.

**Character 22**. General Cornual plate morphology: **(0)** lateral (external) and posterior sides convex, **(1)** lateral (external) side concave and posterior side convex, **(2)** lateral (external) side convex and posterior side concave, **(3)** all sides rounded/convex so appears triangular.

**Character 23**. Lateral projection of cornual plates: **(0)** lateral projection or cornual plate less than brachial plate, **(1)** lateral projection the same as brachial plates, **(2)** lateral projection of cornual just up to greater than double that of the branchial plates **(3)** lateral projection vastly greater than brachial plate **(4)** cornual plates placed on dorsal shield.

**Character 24**. Posterior extension of cornual plates: **(0)** less than posterior margin of dorsal plate, **(1)** equal to posterior margin of dorsal plate, **(2)** greater than posterior margin of dorsal plate.

**Character 25**. Ornamentation of cornual plates: **(0)** scale like ornamentation, **(1)** long ridges parallel to the lateral (external) edge.

**Character 26**. Dorsal plate (separate): **(0)** absent, **(1)** present.

**Character 27**. Dorsal shield: **(0)** absent, **(1)** present. Dorsal shield composed or the equivalent to the dorsal plate, orbital plates, pineal plates and rostral plate. Present condition seen in cyathaspids.

**Character 28**. Dorsal plate surrounded by ‘fields of tesserae’: **(0)** absent, **(1)** present.

**Character 29**. Posterior margin of dorsal plate: **(0)** sinuous medial peak, **(1)** medial peak, **(2)** straight, **(3)** sinuous, **(4)** rounded.

**Character 30**. Brachial notch in dorsal plate: **(0)** absent, **(1)** present. Notch in the dorsal shield for brachial opening.

**Character 31**. Embayment in dorsal plate to accommodate the cornual plates: **(0)** absent, **(1)** present.

**Character 32**. Medial posterior process (posteriorly directed medial peak/extension of the dorsal plate): **(0)** absent, **(1)** present.

**Character 33**. Notch at posterior end of the dorsal plate: (to house the dorsal spine or dorsal fulcral scale, can be enclosed), **(0)** absent, **(1)** present.

**Character 34**. Dorsal spine: **(0)** absent, **(1)** present.

**Character 35**. Separate dorsal spine: **(0)** absent, **(1)** present. Dorsal spine is its own separate plate not fused to the dorsal shield as in some Cyathaspididae.

**Character 36**. Dorsal spine base enclosed by dorsal plate: **(0)** absent, **(1)** present.

**Character 37**. Ornamentation of dorsal spine: **(0)** scale like, **(1)** longitudinal ridges.

**Character 38**. Orientation of the mouth: **(0)** ventral position, **(1)** dorsal position.

**Character 39**. Number of pairs of lateral postoral (orogonal) plates: **(0)** 1 pair, **(2)** 2 pairs.

**Character 40**. Accessory plates along dorsal plate margin: **(0)** absent, **(1)** present.

**Character 41**. Internal organ impression: **(0)** absent, **(1)** present.

**Character 42**. Continuous tubercle bands (ornamentation) pattern on dorsal shield: **(0)** absent, **(1)** present.

**Character 43**. Arrangement of dentine tubercle ridges on dorsal plate: **(0)** lateral addition of dentine ridges, **(1)** concentric circles from a primordium.

**Character 44**. Position of primordium for concentric growth on dorsal plate: **(0)** primordium in the anterior half of the dorsal plate, **(1)** primordium in the centre of dorsal plate**,** **(2)** primordium in the posterior half of the dorsal plate.

**Character 45**. Dentine tubercle edge ornamentation: **(0)** smooth, **(1)** crenulate, **(2)** serrated.

**Character 46**. Tops of dentine ridges: **(0)** smooth, **(1)** crested.

**Character 47**. Ornamentation configuration within the banding: **(0)** uniform (continuous ridges), **(1)** undulating ridges (tuberculated ridges), **(2)** rows of tubercles, **g** tubercles not in rows.

**Character 48**. Anterior end of supra-orbital canal (SOC), **(0)** convergent, **(1)** parallel, **(2)** divergent.

**Character 49**. Middle/Posterior end of SOC, **(0)** convergent, **(1)** parallel, **(2)** divergent.

**Character 50**. Posterior prolongation of SOC, **(0)** between orbital and pineal plate/areas, **(1)** onto the pineal plate/area, **(2)** onto orbital plate/area.

**Character 51**. Position of SOC, **(0)** medial, **(1)** lateral.

**Character 52**. Anterior extension of Median dorsal canal (MDC) when meeting the pineal canal, **(0)** pineal plate, **(1)** orbital plate, **(2)** meets pineal canal on the dorsal plate, **(3)** between orbital and pineal plates.

**Character 53**. Anterior end of MDC, **(0)** convergent, **(1)** parallel, **(2)** divergent.

**Character 54**. Anterior MDC begins on the dorsal disc without connection to any other canals, **(0)** absence, **(1)** presence.

**Character 55**. Pineal canal, **(0)** absence, **(1)** presence.

**Character 56**. Pineal canal, **(0)** loops around pineal plate, **(1)** loops through pineal plate.

**Character 57**. Number of transverse commissures (TC) on the dorsal plate **(0)** 3 **(1)** 4.

**Character 58**. TC pattern, **(0)** radial, **(1)** parallel, **(2)** anterior set parallel, posterior 2 sets radial, **(3)** anterior 2 sets parallel, posterior radial.

**Character 59**. Pattern of first pair (anterior) of TC, **(0)** straight, **(1)** concave.

**Character 60**. Pattern of second pair (middle) of TC, **(0)** straight, **(1)** concave, **(2)** convex. Contingent on ch.58(0).

**Character 61**. Pattern of third pair of TC (posterior), **(0)** straight, **(1)** concave, **(2)** convex. Contingent on ch.58(0).

**Character 62**. 2 pairs of continuous median transverse commissures (MTC), **(0)** absence, **(1)** presence.

**Character 63**. First pair of MTC **(0)** continuous from TC, **(1)** anterior to TC.

**Character 64**. Second pair (middle) of TC contact with lateral dorsal canal (LDC) in relation to contact with MDC in radial canal forms, **(0)** anterior, **(1)** parallel, **(2)** posterior. Contingent on ch.58(0).

**Character 65**. First pair (anterior) TC contact with LDC **(0)** anterior third or LDC, **(1)** median third of LDC. Contingent on ch.58(0).

**Continuous Characters**

**Character 1**. Ratio of pineal plate width to pineal plate length (character 66 in Randle & Sansom, 2017).

**Character 2**. Ratio of pineal plate width to orbital plate median lamellae length (character 67 in Randle & Sansom, 2017).

**Character 3**. Ratio of rostral plate width to rostral plate length (character 68 in Randle & Sansom, 2017).

**Character 4**. Ratio of rostral plate length to dorsal plate length (character 69 in Randle & Sansom, 2017).

**Character 5**. Ratio of the median process of the orbital plate length to distance from orbital opening to orbital opening (character 70 in Randle & Sansom, 2017).

**Character 6**. Ratio of posterior process of orbital plate length to orbital plate length (character 71 in Randle & Sansom, 2017).

**Character 7**. Ratio of anterior process of orbital plates length to orbital plate length (character 72 in Randle & Sansom, 2017).

**Character 8**. Ratio of orbital plate median lamellae length to orbital plate length (character 73 in Randle & Sansom, 2017).

**Character 9**. Ratio of orbital plate length to dorsal plate length (character 74 in Randle & Sansom, 2017).

**Character 10**. Ratio of the distance of the branchial opening from the anterior end of the dorsal plate to dorsal plate length (character 75 in Randle & Sansom, 2017).

**Character 11**. Ratio of brachial plate length to dorsal plate length (character 76 in Randle & Sansom, 2017).

**Character 12**. Ratio of cornual plate length to dorsal plate length (character 77 in Randle & Sansom, 2017).

**Character 13**. Ratio of dorsal plate width to dorsal plate length (character 78 in Randle & Sansom, 2017).

**Character 14**. Ratio of dorsal shield width to dorsal shield length (excluding cornual plates) (character 79 in Randle & Sansom, 2017).

**Character 15**. Ratio of distance to widest part of the dorsal plate from the anterior end of dorsal plate to dorsal plate length (character 80 in Randle & Sansom, 2017).

**Character 16**. Ratio of distance to beginning of embayment (in dorsal plate) area from anterior end of dorsal plate to length of dorsal plate (character 81 in Randle & Sansom, 2017).

**Character 17**. Ratio of distance to narrowest part of embayment (in dorsal plate) from the anterior end of the dorsal plate to dorsal plate length (character 82 in Randle & Sansom, 2017).

**Character 18**. Ratio of narrowest part of embayment (in dorsal plate) width to dorsal plate width (character 83 in Randle & Sansom, 2017).

**Character 19**. Ratio of pineal notch depth in dorsal plate to dorsal plate length (character 84 in Randle & Sansom, 2017).

**Character 20**. Ratio of dorsal spine base width to dorsal spine base length (character 85 in Randle & Sansom, 2017).

**Character 21**. Ratio of dorsal spine base length to dorsal plate length (character 86 in Randle & Sansom, 2017).

**Character 22**. Density of ornamentation ridges per mm in medial area of dorsal plate (character 87 in Randle & Sansom, 2017).

**References**

Randle E, Sansom RS. 2017. Exploring phylogenetic relationships of Pteraspidiformes heterostracans (stem-gnathostomes) using continuous and discrete characters. *Journal of Systematic Palaeontology* **15(7)**:583–599.
